# Can Targeting the Sodium Site via Water Molecules Lead to the Development of Safer Opioids?

**DOI:** 10.1021/acscentsci.4c01105

**Published:** 2024-08-08

**Authors:** Daniel Wacker, Marta Filizola

**Affiliations:** Department of Pharmacological Sciences, Icahn School of Medicine at Mount Sinai, New York, New York 10029-6574, United States

The μ-opioid receptor (μOR), a member of the G protein-coupled receptor (GPCR) family, continues to be a key target for pain management and opioid use disorder (OUD), with several μOR agonists still considered the gold standard for effective analgesics and OUD treatments.^1^ However, the numerous serious adverse effects associated with these drugs, including—but not limited to—the respiratory depression linked to the alarming statistics of the opioid epidemic,^2^ underscore the urgent need for safer analgesics and for addressing the chronic and relapsing nature of OUD. To reduce the side effects associated with μOR activation while maintaining its beneficial effects, drug discovery has focused on modulating μOR activity to engage different pathways with distinct efficacies and potencies. However, considerable ambiguity remains about which pathways are linked to specific (patho)physiological outcomes, and even promising preclinical effects in mice have so far failed to translate into safer medications devoid of any liability.^3^ Novel tools are therefore needed to complement existing chemical probes that bind at the orthosteric site, where endogenous opioid ligands bind. Of particular interest are tools that can leverage the potential of allosteric sites, such as the highly conserved allosteric binding site of sodium ions (Na^+^). In this issue of *ACS Central Science*, Majumdar, McLaughlin, Wang, Hüttenhain, and colleagues propose a new strategy to generate safer opioid-based analgesics. Specifically, the authors report on the development of novel fentanyl derivatives, best exemplified by RO76, which engage residues of the Na^+^ site within the μOR via a water-mediated interaction.^4^ Along with their precursor lead analog, C6 guano, these fentanyl derivatives introduce a novel class of tools to the existing arsenal, as they achieve distinct pharmacological activities by extending to the allosteric Na^+^ site from their main μOR orthosteric binding site.

The differential modulation of opioid agonists and antagonists by Na^+^ has been known for more than half a century.^5^ The collapsed Na^+^ binding site and key protonated (neutral) residue D114^2.50^ in active μOR and other GPCR structures likely contribute to a weaker binding affinity of Na^+^ ions. This allows Na^+^ to more easily translocate through the receptor and egress from the cytosol in an active receptor compared to an inactive one, as we demonstrated a few years ago using a combination of tens of microseconds of standard molecular dynamics (MD) and umbrella sampling simulations, Markov State Models, and machine learning tools.^6^

In the pursuit of developing novel opioid tools, the authors not only generate new bitopic ligands that interact with both orthosteric and allosteric sites of μOR, but also employ an interesting strategy in which Na^+^ binding site residues are engaged indirectly through water molecules. Given the conservation of this site in other class A GPCRs, the implications of the design and pharmacological activities extend far beyond opioid receptors. Exploiting the Na^+^ binding site of other GPCRs, with or without water-mediated interactions, could in principle be applied to develop pathway-selective drugs for a variety of therapeutic targets, potentially revolutionizing the treatment of numerous conditions.

**Figure 1:**
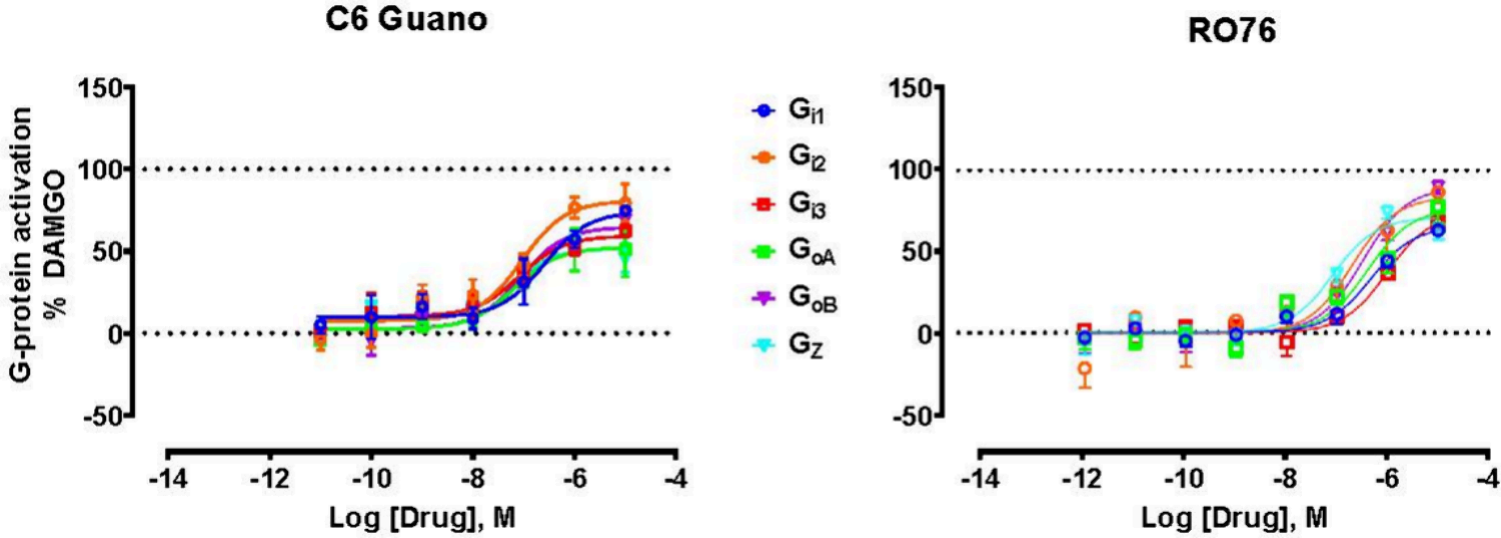
μOR-mediated Gα subtype selectivity profiles induced by C6 guano or RO76 using the Trupath assay. RO76 data were reproduced with permission from ref (4). Copyright 2024 American Chemical Society. C6 guano data were reproduced with permission from ref (7). Copyright 2024 Springer Nature.

The design of RO76 was motivated by the discovery that its precursor lead analog C6 guano exhibited μOR-dependent antinociception with reduced adverse effects compared to clinically used opioids but had limited therapeutic potential due to poor blood-brain barrier (BBB) penetration. Unlike RO76, which exhibits a partial agonism profile at all Gα_i/o/z_ subtypes and low recruitment of both β-arrestin subtypes *in vitro*, C6 guano interacts directly with D114^2.50^ in the Na^+^ site and shows reduced activation of Gα_o_ and Gα_*z*_ compared to Gα_i_^7^ (Figure 1). Encouragingly, RO76 is reported to show better BBB penetration while maintaining analgesic properties with reduced side effects compared to morphine in animal models. These pharmacological distinctions are rationalized as the likely result of the different interactions in the Na^+^ site. However, it remains unknown how the interactions of C6 guano and RO76 in the Na^+^ site result in these different activities. The authors propose that the indirect contact with D114^2.50^ through a single water molecule is responsible for RO76’s distinct pharmacology. However, MD studies reveal that this indirect contact is only formed in ∼20% of cases, requiring further clarifying studies. As lower intrinsic activity has recently emerged as a key strategy to reduce μOR-related side effects,^8^ and the precise G protein-mediated pathways governing therapeutic and adverse outcomes remain understudied,^9−12^ RO76 is a valuable new tool to investigate the precise pathways and activation thresholds required to address opioid safety.

Despite these promising findings, several challenges and opportunities lie ahead. First and foremost, what are the mechanisms by which different interactions in the Na^+^ site promote different signaling outputs, and could such insights lead to the rational design of pathway-selective probes via direct/indirect interaction with distinct Na^+^ site residues? While RO76 shows favorable in vivo activity, its lower in vitro potency compared to other analogs indicates that further optimization is needed. This optimization may or may not require G-protein selectivity for optimal therapeutic outcomes. This selectivity is lost with RO76, suggesting that water-mediated and direct interactions with residues of the Na^+^ binding site may lead to the activation of different G protein pathways. Another challenge is understanding the mechanisms underlying the reduced respiratory depression and physical dependence observed with RO76. Initial findings suggest that targeting the Na^+^ site indirectly through water molecules may modulate signaling pathways differently from traditional opioids. More research is needed to fully elucidate these mechanisms, including further investigating the role of proximal proteins like putative phosphatase Paladin 1 (PALD1) in μOR signaling, as highlighted by the authors. Additionally, since RO76 is derived from fentanyl, it will be important to compare its *in vivo* effects directly to those of fentanyl to better understand and contrast the underlying mechanisms. Future research could also explore the potential of targeting Na^+^ sites in other GPCRs. The rational design of ligands that exploit structural waters in the sodium binding pockets of these receptors could lead to the development of pathway-selective drugs with improved therapeutic profiles. This requires a multidisciplinary approach, combining medicinal chemistry, structural biology, and computer simulations to identify and optimize new ligands.

In summary, this paper represents a significant advancement in the field of GPCR research. By targeting the Na^+^ site through water molecules, the authors have developed a novel bitopic ligand with a potential toward the development of promising therapeutics. Although challenges remain, the insights gained from this study provide a strong foundation for future research aimed at developing safer and more effective opioid drugs. Moreover, this strategy can be adopted to target other GPCRs, providing complementary tool compound sets to study a wide variety of mechanisms in health and disease. The research community should build on these findings, embracing the innovative strategies and interdisciplinary collaboration needed to push the frontiers of drug discovery even further.
